# Posture Tracking of Active Capsule Endoscopes Integrated with Magnetic Actuation Using Hall-Effect Sensors

**DOI:** 10.3390/mi17030327

**Published:** 2026-03-05

**Authors:** Junho Han, Kim Tien Nguyen, Eui-Sun Kim, Jong-Oh Park, Eunho Choe, Chang-bae Moon, Jayoung Kim

**Affiliations:** 1 Department of Mechanical Engineering, Chonnam National University, Gwangju 61186, Republic of Korea; hjh0426@jnu.ac.kr; 2 Korea Institute of Medical Microrobotics, Gwangju 61011, Republic of Korea; nkt@kimiro.re.kr (K.T.N.); kes@kimiro.re.kr (E.-S.K.); jop@kimiro.re.kr (J.-O.P.); 3 Department of Biosystems Engineering, Chungbuk National University, Cheongju 28644, Republic of Korea

**Keywords:** capsule endoscopy, magnetic localization, hall-effect sensor, position and orientation estimation, magnetic actuation

## Abstract

A capsule endoscope (CE) provides noninvasive access to the gastrointestinal tract, offering diagnostic information that cannot be obtained through external imaging alone. However, during the examination inside the stomach, the CE’s posture may change rapidly as it moves within a dynamically deforming organ, making it difficult to determine its orientation using only the onboard camera feedback. To address this problem, this study proposes a method that employs an external array of Hall Effect Sensors (HES) to estimate the capsule’s position and orientation in real time, based on the magnetic field generated by a permanent magnet (PM) embedded inside the capsule, without the need for any additional internal sensors. This approach introduces a unified magnetic actuation and localization framework that enables real-time 5-degree-of-freedom posture estimation using only the internal PM of the capsule. Furthermore, the proposed system features an integrated architecture capable of simultaneous actuation and localization. To enhance system practicality, the sensor module and communication board were combined into a single unit that employs a digital serial communication scheme, eliminating the need for analog to digital conversion of sensing signals. By avoiding additional onboard sensors and employing a PM-based actuation system, the proposed system simplifies hardware configuration by preserving capsule miniaturization and by eliminating the high power consumption and thermal issues associated with electromagnet-based actuation, while maintaining accurate real-time tracking performance. Through an optimization process, the system achieved a position error of less than 2 mm and an angular error within 2° over a sensing range of up to 60 mm. Repeated experiments further validated the system’s effectiveness and reliability under realistic operating conditions, demonstrating its feasibility for compact and clinically applicable active capsule endoscopy systems.

## 1. Introduction

CE is an innovative medical technology developed for the diagnosis of gastrointestinal (GI) disorders. It is encapsulated in a compact and ingestible form factor incorporating a miniature camera that captures and transmits real-time images of the GI tract. These images provide clinicians with direct visual access to the internal mucosa, thereby supporting accurate diagnosis and facilitating the formulation of appropriate treatment strategies. For effective clinical application of CE, accurate posture tracking of both position and orientation is essential. During the examination, the capsule’s spatial location may change rapidly due to variations in patient posture and internal organ motion. When the capsule is in the stomach, determining its posture solely from camera images is highly challenging. This issue becomes more pronounced in actively controlled capsules, where precise posture tracking is indispensable to improving navigational accuracy and ensuring effective system controllability. Regardless of whether the capsule is operated manually or through computer-assisted control, accurate posture information is imperative for steering it toward the intended target with minimal effort. Accurate posture tracking not only facilitates efficient navigation but also plays a vital role in enhancing diagnostic precision and optimizing treatment planning.

To meet this need, various posture tracking techniques have been investigated, including various approaches based on radio frequency (RF), ultrasound, medical imaging, and inertial measurement unit (IMU). In RF-based tracking methods, the generated RF signals can penetrate biological tissues, thereby enabling posture estimation. However, these methods demand precise synchronization between transmitters and receivers as well as a stable wireless communication link. Hence, system complexity and maintenance burden are increased. Moreover, RF-based methods are highly susceptible to multipath effects and environmental interference, which often result in substantial tracking errors [1,2]. Other modalities also present inherent limitations that restrict their practical applicability. Ultrasound-based tracking requires dedicated transducers and may experience reduced accuracy due to reflection and absorption phenomena [3,4]. Image-based methods utilizing computed tomography (CT) or magnetic resonance imaging (MRI) can deliver high resolution posture information. Nevertheless, they are generally impractical for real time tracking owing to acquisition and processing delays. In addition, CT involves exposure to ionizing radiation, whereas MRI necessitates expensive equipment and complex infrastructure [5]. IMU-based tracking is relatively robust against environmental perturbations but suffers from cumulative drift over time and thus generally requires integration with complementary sensing modalities [6,7,8]. A summarized comparison of these representative localization approaches is provided in Table 1.

To overcome the limitations of existing techniques, magnetic field-based posture tracking methods have been actively investigated for CE applications. Several studies have proposed approaches that estimate the position and orientation of a capsule by measuring the magnetic field generated by a PM embedded inside the capsule using an external HES array. In some studies, five-degree-of-freedom (5-DoF) posture estimation was achieved under electromagnet-based actuation environments by introducing magnetic field model compensation or higher-order differential signal processing techniques to suppress strong magnetic interference [9,10]. However, a number of these approaches primarily focused on localization performance evaluation and thus exhibited limitations in simultaneously demonstrating real-time posture estimation and active capsule actuation within dynamic experimental scenarios. Recently, mobile electromagnet–sensor integrated systems have been reported to secure large workspaces and to demonstrate simultaneous real-time actuation and localization [11]. Nevertheless, these electromagnet-centered approaches often involve large system footprints and high power consumption, which limit their applicability to CE applications where miniaturization and energy efficiency are critical.

This study adopts an actuation scheme based on external PMs. The capsule contains only a PM, while a fixed external HES array is employed to estimate the capsule posture in real time with 5-DoF. The HES array measures the spatial distribution of magnetic flux density variations induced by the position and orientation of the embedded magnet, and the acquired measurements are reconstructed into the capsule posture using a nonlinear least-squares estimator based on the Levenberg–Marquardt (LM) algorithm. The proposed localization method utilizes the full spatial magnetic field distribution captured by a multi-sensor array and reconstructs both position and orientation simultaneously through a physics-based dipole model. By formulating the posture estimation as a nonlinear optimization problem, the method ensures consistent coupling between position and orientation parameters while maintaining real-time computational feasibility. In addition, offset compensation for the external actuation field is incorporated into the estimation process, enabling stable posture reconstruction even during active magnetic actuation. Because the actuation magnetic field is provided exclusively by the external PM, the proposed system fundamentally eliminates magnetic interference, high power consumption, thermal issues, and hardware complexity associated with electromagnet-based systems. As a result, the proposed approach achieves capsule miniaturization and simplified system architecture without requiring additional onboard transmitters or sensors, while enabling stable and unified realization of capsule actuation and posture estimation.

**Figure 1:**
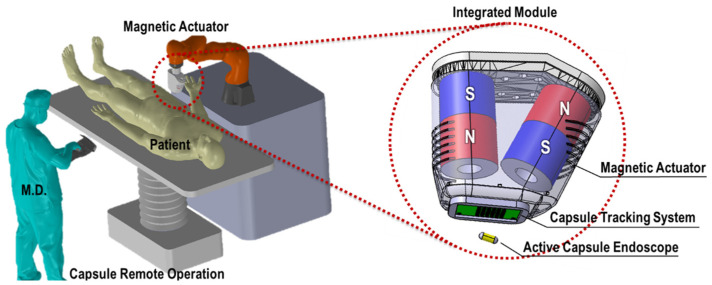
Schematic of the proposed magnetic field-based tracking system, consisting of an HES array with a magnetic actuator.

Figure 1 shows the schematic diagram of the proposed magnetic field-based tracking system. A sensor module consisting of 40 HES was designed with a spacing of 10 mm, covering an area of 70 × 40 mm^2^. Two PMs were attached to the end effector of a robotic arm at different tilt angles, and the HES array was located beneath them, enabling real-time measurement of magnetic field variations during capsule actuation. By integrating the magnetic actuator with the HES array, capsule’s position and orientation can be simultaneously controlled and estimated, enabling stable and continuous localization without the need for complex hardware configurations [12,13].

In addition, the sensor data acquisition architecture was designed to digitize the analog outputs of the HES directly on the board and transmit them via serial communication, thereby enhancing scalability and commercial applicability. Conventional analog data acquisition systems that collect voltage signals directly become increasingly complex and susceptible to noise as the number of sensors increases. To overcome this limitation, the HES board integrates 24-bit ADCs with the communication board, enabling stable transmission of 40-channel sensor data through RS-422-based digital serial communication. This configuration not only simplifies the overall hardware design but also enhances the reliability and scalability of the system.

## 2. Materials and Methods

### 2.1. Localization Method Using HES

**Figure 2:**
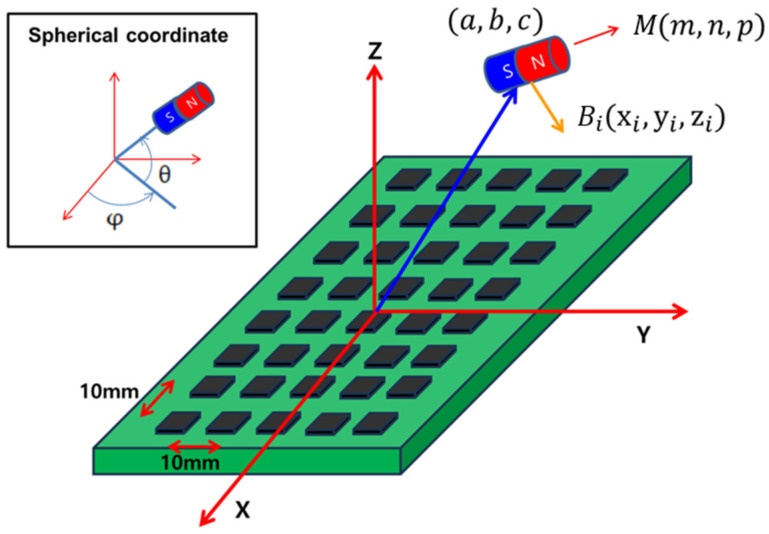
HES array and magnet diagram.

A neodymium magnet with a diameter of 9 mm and a thickness of 5 mm was embedded inside the CE. This magnet serves not only as a magnetic source for localization but also interacts with the external magnetic system to enable active actuation. In this study, the magnetic field of the magnet inside CE was measured using the external HES array to estimate its position and orientation. A magnetic dipole model was employed, with the magnet’s position denoted as $\left(a,b,c\right)$ and its orientation vector as $\vec{M}=\left(m,n,p\right)$. The location of each HES is represented by $\left(x_{i},y_{i},z_{i},\;i\;=\;1,\;2,\;\ldots \;N\right)$. According to the magnetic dipole model, the magnetic field $\vec{B_{i}}$ induced at the *i*-th sensor can be expressed as Figure 2.

(1) $$\vec{B_{i}}=B_{T}\left(\frac{3\left(\vec{M}\;\cdot \;\vec{R_{i}}\right)\vec{R_{i}}}{R_{i}^{5}}-\frac{\vec{M}}{R_{i}^{3}}\right)$$

where $B_{T}$ is a scalar coefficient related to the magnetic dipole moment of the magnet, $\mu_{r}$ is the relative permeability of the PM, $\mu_{0}$ is the vacuum permeability of free space, and $M_{T}$ denotes the magnetization amplitude of the magnet.

(2) $$B_{T}=\frac{\mu_{r}\mu_{0}M_{T}}{4\pi }$$

The vector $\vec{R_{i}}$ points from the magnet to the sensor and is given by

(3) $$\vec{R_{i}}=\left(x_{i}-a,\;y_{i}-b,\;z_{i}-c\right)$$

(4) $$R_{i}=\sqrt{{{(x}_{i}-a)}^{2}+{{(y}_{i}-b)}^{2}+{{(z}_{i}-c)}^{2}}\;for\;i=1,2,\ldots ,N$$

To explicitly parameterize the magnet’s orientation during the optimization process, the orientation vector $\vec{M}=\left(m,n,p\right)$ can also be represented in the spherical coordinates, where the angles θ and φ correspond to the CE’s orientation parameters to be estimated.

(5) $$m=\sin\left(\theta \right)\cos\left(\varphi \right),\;n=\sin\left(\theta \right)\sin\left(\varphi \right),\;p=\cos\left(\theta \right)$$

The components of the magnetic flux density in each axis direction are expressed as

(6) $$\begin{array}{c} B_{x,i}=B_{T}\left(\frac{3S_{i}\cdot \left(x_{i}-a\right)}{R_{i}^{5}}-\frac{m}{R_{i}^{3}}\right)\\ B_{y,i}=B_{T}\left(\frac{3S_{i}\cdot \left(y_{i}-b\right)}{R_{i}^{5}}-\frac{n}{R_{i}^{3}}\right)\\ B_{z,i}=B_{T}\left(\frac{3S_{i}\cdot \left(z_{i}-c\right)}{R_{i}^{5}}-\frac{p}{R_{i}^{3}}\right)\\ S_{i}=m\left(x_{i}-a\right)+n\left(y_{i}-b\right)+p\left(z_{i}-c\right) \end{array}$$

The estimation of the magnet’s 5-DOF posture is formulated as a nonlinear least-squares optimization problem. The pose parameter vector is defined as $p=[a,b,c,\theta ,\phi {]}^{T}$. The residual vector is defined as

(7) $$f\left(p\right)=B_{meas}-B_{model}\left(p\right)$$

where $B_{meas}\in R^{3N}$ denotes the stacked magnetic flux density vector measured by all N sensors, and $B_{model}(p)\in R^{3N}$ represents the corresponding theoretical magnetic flux density vector computed using the magnetic dipole model. The optimal pose parameters are obtained by minimizing the squared norm of the residual vector:

(8) $$\underset{p}{\min}{\left\|f\left(p\right)\right\|}^{2}$$

In this study, the Levenberg–Marquardt algorithm was employed, as it is suitable for problems with small residuals.

### 2.2. Magnetic Field Analysis for Sensor Placement

Figure 1 shows the configuration of the HES module positioned beneath the PM of the magnetic actuator, which is designed to support simultaneous magnetic actuation and localization of the CE. As the distance between the capsule and the PM increases, the magnetic actuation performance decreases. Therefore, the HES module should be placed as close to the PM as possible. At the same time, it is necessary to determine an installation position that satisfies the magnetic field condition to prevent the HES from reaching magnetic saturation. For this purpose, the magnetic field around the PM was measured using a Gauss meter, and the magnetic field strength was analyzed for candidate sensor positions at various vertical distances.

**Figure 3:**
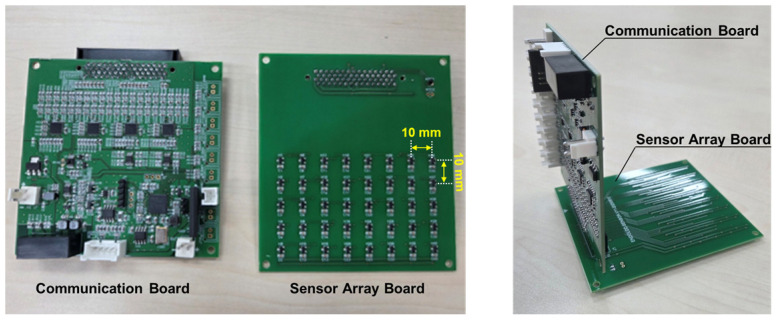
A captured image of the fabricated HES array used in this work.

Figure 3 illustrates the sensor and communication board. The sensing module was constructed using single-axis analog-output HES (DRV5055A2, Texas Instruments, Dallas, TX, USA). The sensors employed in this study were commercially purchased silicon-based HES, which provides a linear operating range of ±42 mT and a sensitivity of 50 mV/mT at a 5 V supply voltage according to the manufacturer’s specifications. The sensor board consists of 40 sensors arranged in an 8 × 5 array with 10 mm spacing over an area of 70 × 40 mm^2^. All the 40 sensors were mounted on a printed circuit board to maintain fixed spatial alignment and structural stability of the sensor module.

Although increasing the number of HES can theoretically enlarge the region of interest (ROI) and improve localization accuracy, expanding the sensing plate introduces practical constraints. A larger sensor plate occupies excessive space beneath the robotic arm, potentially restricting its range of motion or increasing the risk of mechanical interference. This may hinder smooth capsule actuation. Furthermore, when sensors are positioned directly below the external PM, the strong magnetic field can saturate the HES. Therefore, extending the HES array beyond a certain area does not effectively increase the usable sensing region, as portions of the array would operate in a saturated state. In this context, an excessively wide sensing plate provides limited practical benefit. Accordingly, the proposed system adopts an 8 × 5 rectangular arrays with 10 mm spacing to maximize spatial resolution within the mechanically constrained ROI beneath the robotic platform. This configuration assumes that the capsule predominantly moves within the predictable region below the external PM.

The analog signals output from the sensor board are converted into digital signals by a built-in 24-bit A/D converter (ADS1261, Texas Instruments, USA), and the converted data are transmitted via RS-422 communication through the MCU. This configuration allows real-time transmission and storage of 40-channel HES data. The sensor and communication boards are vertically combined and installed below the PMs of the magnetic actuator.

**Figure 4:**
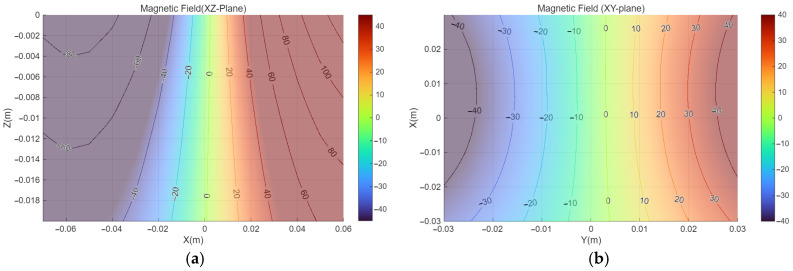
Magnetic field from the magnetic actuator measured by a Gauss meter. (**a**) XZ-plane at y = 0. (**b**) XY-plane at z = 20 mm.

Figure 4 shows the magnetic field distribution generated by the PMs of the magnetic actuator. Figure 4a presents the magnetic field in the XZ-plane along the central axis of the PM, while Figure 4b shows the magnetic field in the XY-plane measured at a vertical distance of 20 mm, corresponding to the installation gap of the sensor board.

**Figure 5:**
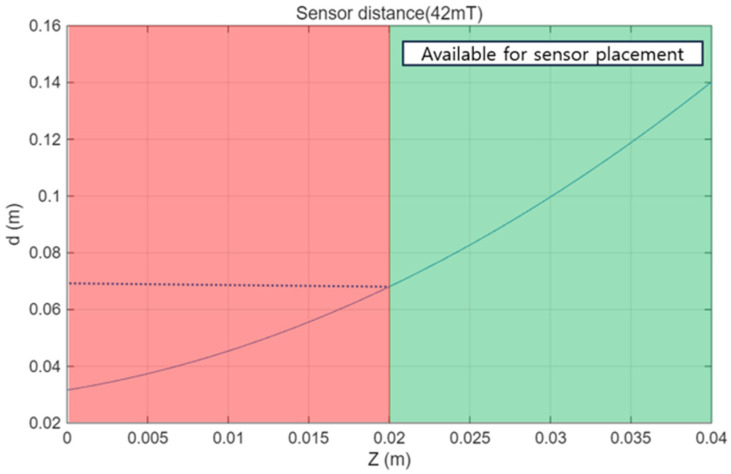
Effective sensor placement width d as a function of the installation gap between the magnetic actuator and the HES module.

Figure 5 presents the relationship between the installation distance z between the sensor module and the external PM and the effective sensor placement width d. Here, the effective placement width d is defined as the maximum lateral width of the sensor module within which the magnetic flux density remains below 42 mT, corresponding to the upper linear operating limit of the employed HES. The contour shown in Figure 5 corresponds to an iso-field line at 42 mT, which represents the sensor saturation threshold. The HES module consists of an 8 × 5 sensor array with a spacing of 10 mm, resulting in a total module width of 70 mm. Therefore, to ensure that all sensors operate within the linear range, the condition d≥70 mm must be satisfied. Based on this criterion, the minimum allowable installation distance z that satisfies d≥70 mm was identified. Since reducing the distance between the external PM and the sensor module is beneficial for magnetic actuation performance, the installation distance was selected as the minimum value that avoids sensor saturation while keeping full sensor coverage. This design choice enables simultaneous magnetic actuation and localization without compromising sensor linearity.

The magnetic field distribution at the selected gap of 20 mm is shown in Figure 4b. Near the edge of the sensor array, where the distance from the PM pole is shorter, the magnetic field reached approximately ±50 mT, resulting in partial saturation of some sensors. Even when saturated sensors were included, interpolation based on the outputs of adjacent unsaturated sensors were applied to estimate the overall magnetic field distribution. Through this approach, the influence of the limited saturation region was minimized, enabling stable magnetic field measurement and position estimation for the entire sensor array.

## 3. Experimental Result

### 3.1. Experimental Setup

**Figure 6:**
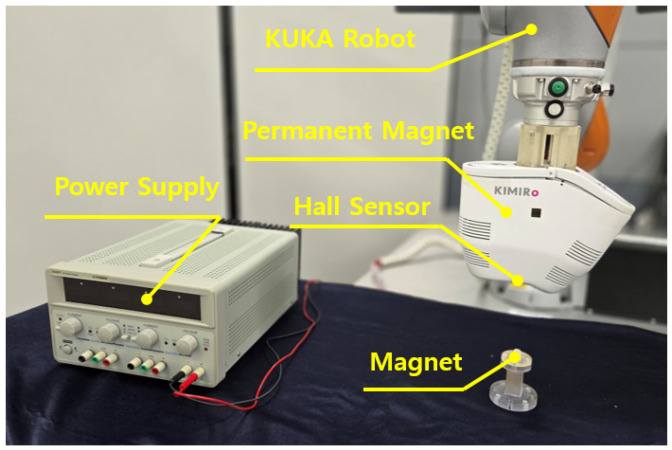
Experimental setup for active capsule posture tracking.

The experimental system was configured as shown in Figure 6. To achieve miniaturization for simultaneous actuation and localization of the CE, a cylindrical neodymium magnet of grade N52 (diameter 9 mm, thickness 5 mm) was embedded inside the capsule. The system consisted of a power supply (E3005T DC Power Supply, VUPOWER, Daejeon, Republic of Korea), a control PC, a robotic arm for position control (KUKA LBR iiwa 7 R800, KUKA Robotics, Augsburg, Germany), and a pair of external N52 PMs used for magnetic actuation (radius 35 mm, height 60 mm). The robotic arm was controlled via LabVIEW 2025Q1, and the position estimation algorithm was implemented in MATLAB R2024a. In the experimental configuration, the distance between the capsule magnet and the HES array was selected such that the observation region lay outside the near field of the magnet, where the magnetic dipole approximation provides sufficient accuracy. Previous studies have shown that for cylindrical PMs, the dipole model yields negligible error when the sensing distance is several times larger than the characteristic dimensions of the magnet [14]. Magnetic field data acquired from the HES array were processed at an update rate of 10 Hz, allowing real-time estimation of the capsule position and orientation throughout the experiments.

### 3.2. Experimental Results

As described in Figure 1, the HES module was attached to the lower part of the PM in the magnetic actuator mounted on the end effector of the robotic arm, whereas the small PM intended for insertion into the CE was fixed to the base. The robotic arm was then moved to measure the magnetic field data obtained from the HES. In this setup, the HES senses the combined magnetic field generated by both the PMs in the magnetic actuator and the CE. Since the localization algorithm must utilize only the magnetic field generated by the capsule magnet, the magnetic field component induced by the PM of the magnetic actuator was compensated through a calibration process. For this purpose, an initialization procedure was performed in which the magnetic field measured solely from the PM of the magnetic actuator, in the absence of the capsule magnet, was set as the offset value. Subsequently, the offset was subtracted from all measured data to obtain magnetic field values compensated for the unnecessary PM’s influence. Since the HES module and the actuator PM are rigidly fixed on the same mechanical structure, their relative pose remains constant during the experiment. Therefore, the magnetic field contribution from the actuator magnet induces a static bias at each sensor, which remains stable regardless of the capsule motion or robotic arm movement, and no recalibration is required during operation.

**Figure 7:**
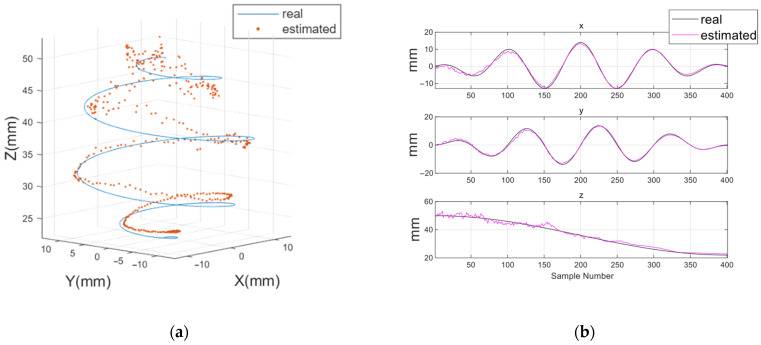
Position estimation results of the capsule. (**a**) Trajectory of the capsule and (**b**) position estimation results obtained from the HES.

**Figure 8:**
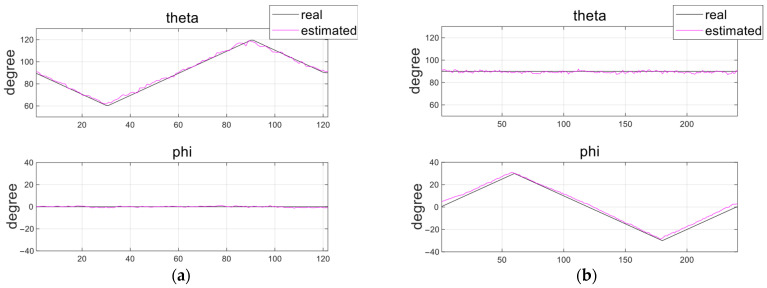
Orientation estimation results of the capsule. (**a**) Pitch angle and (**b**) Yaw angle.

To quantitatively evaluate the localization performance of the HES module, two experiments were conducted: position estimation and orientation estimation. For position estimation, as illustrated in Figure 7a,b, the robotic arm was moved along a spiral trajectory whose radius gradually increased from 0 to 14 mm, descending from a height of 50 mm to 22 mm. During this motion, HES data were collected at a total of 400 measurement points to assess the 3D position estimation accuracy of the proposed system. For orientation estimation, the distance between the magnetic capsule and the HES module was fixed at 50 mm, and the magnet was rotated from −30° to 30° in both the pitch and yaw directions. The angles *θ* (with respect to the z-axis) and *φ* (with respect to the x-axis) in the spherical coordinate system were calculated, and the results are described in Figure 8a,b.

All experiments estimated the capsule’s position and orientation in real time based on the magnetic field data measured from the HES array, and the results were compared with the actual positions obtained from the robotic arm. To verify repeatability, each experiment was performed five times under identical condition.

Table 2, Figure 7 and Figure 8 present estimation results of the capsule posture in numerical and graphical forms. In the position-estimation experiment, the capsule orientation was fixed at reference angles *θ* = 0° and *φ* = 0°. RMSE values for these fixed angles were additionally computed to verify that the estimated orientation remained consistent with the reference. Under this condition, the positional RMSEs were 0.98 mm, 0.76 mm, and 1.62 mm for the x, y, and z coordinates, respectively.

In the orientation-estimation experiment, the relative position between the capsule and the HES module was fixed; therefore, the x, y, and z positions did not vary during angular rotation. While rotating in the *φ* direction, the *θ* angle was maintained at approximately 90° due to magnetic alignment induced by the external PM. As shown in Figure 8, the estimated angles generally followed the reference angle variations with good agreement. RMSE values were calculated using the same procedure, and the RMSEs for the estimated orientation (*θ* and *φ*) were 2.0° and 1.7° in the pitch direction, and 1.9° and 2.1° in the yaw direction. By applying the same RMSE-based evaluation to all degrees of freedom, the results indicate that positional errors remained below 2 mm, while orientation errors were approximately 2°. Because the relative position remained constant during rotation, the magnetic field variation was primarily dominated by angular change, which contributed to stable and consistent estimation performance. These results confirm that the proposed HES-based tracking algorithm can reliably estimate the 5-degree-of-freedom posture of the magnetic capsule.

**Figure 9:**
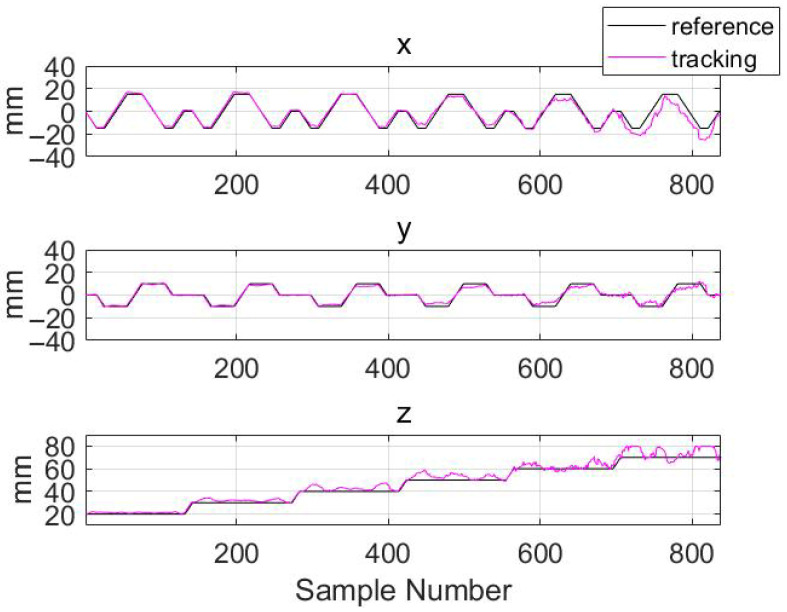
Position estimation results at different heights.

Figure 9 and Table 3 present the experimental results for the detectable localization range. The vertical distance z was increased from 20 mm to 70 mm in 10 mm increments, and at each z level, the capsule was scanned across the x–y plane by moving x and y from −20 mm to 20 mm to form a rectangular trajectory. The localization error increased gradually as the sensing distance increased up to 60 mm, remaining within approximately 2–3 mm. However, at 70 mm, the error increased significantly, making accurate estimation difficult. These results indicate that the practical sensing limit of the proposed system is approximately 60 mm.

To further analyze the modeling behavior of the dipole-based localization method with respect to sensing distance, the relationship between sensing distance and estimation error was examined. The embedded magnet has a diameter of 9 mm, while the sensing distance ranged from 20 mm to 70 mm. Within the 20–60 mm range, the estimation error increased smoothly with distance without abrupt deviation. This consistent trend suggests that the dipole-based magnetic model maintains stable estimation characteristics in this range. The noticeable error increase at 70 mm coincides with the significant reduction in magnetic field magnitude due to the inherent 1/r^3^ decay of dipole fields. Therefore, the degradation in estimation performance at extended distances is primarily associated with reduced signal amplitude and signal-to-noise ratio, rather than with an instability of the dipole modeling assumption itself. These observations support the applicability of the dipole approximation within the practical sensing range of the proposed system [14].

To assess position tracking performance, the capsule was magnetically actuated by external PMs and moved within a 100 × 100 mm planar workspace. During the motion, magnetic field data were continuously acquired using the HES array. The experiments were conducted at two different vertical distances of 20 mm and 50 mm between the capsule and the sensor module to evaluate the position estimation accuracy under varying height conditions. The actual capsule position during motion was simultaneously measured using a camera-based tracking system and used as the reference for quantitative performance evaluation.

**Figure 10:**
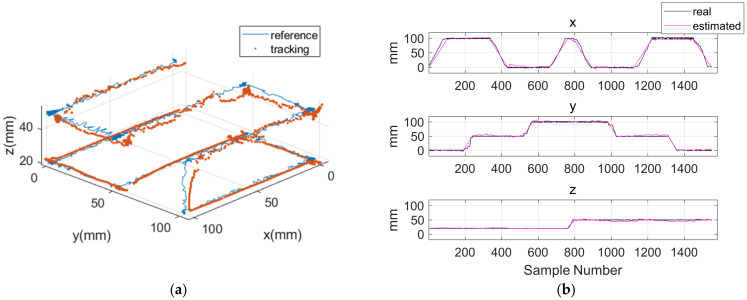
Estimated and Ground Truth Positions of the Magnetically Actuated Capsule. (**a**) 3D Trajectory and (**b**) Axis-wise Position Comparison.

Figure 10 and Table 4 summarize the results of position and orientation estimation during magnetic actuation. The RMSE for position tracking were 6.6 mm, 3.7 mm, and 2.5 mm along the x-, y-, and z-axes, respectively, while the orientation estimation errors were 5.7° and 2.4° for *θ* and *φ*. Compared with the static experiments, a noticeable increase in RMSE was observed under magnetic actuation. This degradation is primarily related to the reference measurement method used during dynamic evaluation. In the static experiments, the capsule was fixed in space, and the robotic arm moved the HES module relative to the capsule. Therefore, the ground-truth pose was defined by the kinematically controlled relative position between the robotic arm and the fixed capsule, providing high positional accuracy. In contrast, during magnetic actuation, the capsule trajectory was measured using a camera-based tracking system. The camera tracks the geometric center of the capsule, whereas the magnetic localization algorithm estimates the position of the embedded magnet, which is located toward the rear side of the capsule. This inherent spatial offset between the tracked capsule center and the actual magnet position introduces additional geometric discrepancy, especially during capsule rotation. As a result, part of the increased RMSE reflects the reference mismatch rather than the intrinsic degradation of the dipole-based localization algorithm itself. Despite this increase, the proposed system maintained continuous and stable pose estimation throughout capsule motion under magnetic actuation.

Although the experiments were conducted under air-based laboratory conditions, the influence of biological tissues should be considered for practical clinical applications. Most biological tissues are weakly diamagnetic, with magnetic susceptibility on the order of 10^−6^, resulting in relative magnetic permeability close to unity ($\mu_{r}$ ≈ 1) [15]. Under static or quasi-static conditions, magnetic fields are therefore expected to experience negligible attenuation or distortion when propagating through biological media. Accordingly, the magnetic dipole-based localization principle adopted in this study is expected to remain approximately valid in tissue environments. Nevertheless, practical factors such as physiological motion, environmental magnetic disturbances, and sensor noise may affect estimation stability and accuracy under vivo conditions. Therefore, further validation using tissue-mimicking phantoms as well as in vivo animal experiments will be necessary to comprehensively evaluate clinical applicability.

## 4. Conclusions

In this study, an HES-based magnetic tracking system was designed and evaluated through both static localization experiments and real-time tracking experiments under magnetic actuation. Under static conditions, the proposed system achieved position estimation errors below 2 mm and orientation errors below 2°, validating the accuracy of the magnetic dipole-based localization algorithm. In addition, real-time tracking experiments were conducted while the capsule was magnetically actuated by external PMs. During these experiments, the capsule motion was continuously tracked using the proposed HES-based system, demonstrating stable and uninterrupted real-time position and orientation estimation during magnetic actuation. These results confirm that the localization algorithm remains operational and robust even under dynamically changing magnetic environments caused by capsule actuation.

Furthermore, by integrating the sensor module and communication board and adopting a digital serial communication scheme, the proposed system eliminates the need for multiple analog signal lines required in conventional A/D conversion architectures. This design simplifies the hardware configuration and enables stable multi-channel data transmission through a single communication interface, thereby enhancing system practicality and commercialization potential. Within the evaluated range, reliable localization performance was maintained when the distance between the magnetic capsule and the HES array was within 60 mm, whereas estimation accuracy degraded significantly beyond 70 mm. In addition, strong external magnetic fields may drive the HES toward saturation, which can degrade measurement accuracy and adversely affect localization performance. Considering that the average human abdominal wall thickness is approximately 30–35 mm, the proposed system provides a sufficient sensing range for typical clinical applications. However, additional magnetic disturbances between the capsule and the HES array may influence measurement stability. Moreover, as the sensing distance increases, the signal-to-noise ratio decreases, which may degrade estimation robustness. Therefore, extending the effective sensing range and improving robustness against magnetic disturbances remain important subjects for future investigations.

The sensing range limitation is primarily governed by the rapid spatial decay of the magnetic dipole field and the resulting signal-to-noise ratio constraints. Although the use of higher-sensitivity sensors could theoretically improve signal detectability at longer distances, such sensors generally exhibit reduced linear operating ranges and are therefore more susceptible to saturation under strong external PM fields used for actuation. Consequently, simple replacement with higher-sensitivity sensing materials does not necessarily guarantee an extended localization range. Instead, an optimal balance among sensor sensitivity, magnetic actuator strength, and signal processing strategies must be considered. Future work will explore system-level approaches, including magnet geometry optimization and advanced signal processing techniques, to expand the effective sensing range.

Overall, the proposed HES-based magnetic tracking system enables real-time estimation of capsule position and orientation using only the magnetic field generated by an internal PM, without requiring additional onboard sensors. The system’s simple and reliable hardware architecture, combined with its real-time tracking capability during magnetic actuation, provides a practical foundation for future integration with magnetic actuation systems and the development of autonomous CE platforms. Future work will focus on developing a hybrid actuation system that combines PMs and electromagnets to enhance controllability and expand the effective workspace of magnetic actuation. In addition, in vivo animal experiments will be conducted to verify system performance under realistic biological tissue environments beyond air-based experimental conditions.

## Figures and Tables

**Table 1:** Comparison of Capsule Localization Methods.

Category	RF-Based	Ultrasound-Based	IMU-Based	Fixed Hall Sensor Array	Proposed Method
Capsule Hardware	RF transmitter	Ultrasound emitter	9-DOF IMU	Permanent magnet	Permanent magnet
External Infrastructure	Antenna array	Ultrasound receivers	None	Fixed Hall sensor plate	HES array
Power Consumption	Moderate	Moderate–High	Low–Moderate	Magnetic inverse modeling	Low (Robot Arm)
Onboard Sensor Requirement	yes	yes	yes	no	no
System Complexity	Antenna array required	Multiple receivers required	Sensor fusion required	Fixed plate geometry	Simplified architecture
Key Advantage	Wireless tracking	Tissue-penetrable	No external array	Simple magnetic model	Capsule miniaturization + low-power integrated actuation & localization

**Table 2:** Estimation Results of Capsule Position and Orientation.

RMSE					
	x (mm)	y (mm)	z (mm)	θ (°)	φ (°)
Position	0.98	0.76	1.62	1.5	0.9
Pitch	1.52	1.07	0.95	2.0	1.7
Yaw	0.88	1.36	1.46	1.9	2.1

**Table 3:** Position Estimation RMSE at Different Heights.

Height (mm)	20	30	40	50	60	70
RMSE (mm)	1.22	1.69	2.33	2.96	2.78	6.23

**Table 4:** Estimation Results of Capsule Position and Orientation under Magnetic Actuation.

RMSE				
x (mm)	y (mm)	z (mm)	θ (°)	φ (°)
6.6	3.7	2.5	5.7	2.4

## Data Availability

The original contributions presented in this study are included in the article. Further inquiries can be directed to the corresponding authors.
